# 3-D Printed Protective Equipment during COVID-19 Pandemic

**DOI:** 10.3390/ma13081997

**Published:** 2020-04-24

**Authors:** Christian Wesemann, Stefano Pieralli, Tobias Fretwurst, Julian Nold, Katja Nelson, Rainer Schmelzeisen, Elmar Hellwig, Benedikt Christopher Spies

**Affiliations:** 1 Department of Orthodontics, Dentofacial Orthopedics and Pedodontics, Charité—Universitätsmedizin Berlin, corporate member of Freie Universität Berlin, Humboldt-Universität zu Berlin, and Berlin Institute of Health, Aßmannshauser Str. 4–6, 14197 Berlin, Germany; 2 Department of Prosthetic Dentistry, Medical Center—University of Freiburg, Center for Dental Medicine, Hugstetter Str. 55, 79106 Freiburg, Germany; stefano.pieralli@uniklinik-freiburg.de (S.P.); julian.nold@uniklinik-freiburg.de (J.N.); benedikt.spies@uniklinik-freiburg.de (B.C.S.); 3 Department of Oral and Maxillofacial Surgery, Medical Center—University of Freiburg, Center for Dental Medicine, Hugstetter Str. 55, 79106 Freiburg, Germany; tobias.fretwurst@uniklinik-freiburg.de (T.F.); katja.nelson@uniklinik-freiburg.de (K.N.); rainer.schmelzeisen@uniklinik-freiburg.de (R.S.); 4 Medical Center—University of Freiburg, Center for Dental Medicine, Department of Operative Dentistry and Periodontology, Faculty of Medicine, University of Freiburg, Hugstetter Str. 55, 79106 Freiburg, Germany; elmar.hellwig@uniklinik-freiburg.de

**Keywords:** 3-D printing, COVID-19, personal protective equipment, face shield

## Abstract

While the number of coronavirus cases from 2019 continues to grow, hospitals are reporting shortages of personal protective equipment (PPE) for frontline healthcare workers. Furthermore, PPE for the eyes and mouth, such as face shields, allow for additional protection when working with aerosols. 3-D printing enables the easy and rapid production of lightweight plastic frameworks based on open-source data. The practicality and clinical suitability of four face shields printed using a fused deposition modeling printer were examined. The weight, printing time, and required tools for assembly were evaluated. To assess the clinical suitability, each face shield was worn for one hour by 10 clinicians and rated using a visual analogue scale. The filament weight (21–42 g) and printing time (1:40–3:17 h) differed significantly between the four frames. Likewise, the fit, wearing comfort, space for additional PPE, and protection varied between the designs. For clinical suitability, a chosen design should allow sufficient space for goggles and N95 respirators as well as maximum coverage of the facial area. Consequently, two datasets are recommended. For the final selection of the ideal dataset to be used for printing, scalability and economic efficiency need to be carefully balanced with an acceptable degree of protection.

## 1. Introduction

The coronavirus (COVID-19) pandemic is challenging healthcare systems worldwide. By the end of January 2020, it was declared an international “public-health emergency” by the World Health Organization (WHO) [1], with the number of COVID-19 patients increasing daily. To ensure the protection of the healthcare workers (HCWs) treating these patients, personal protective equipment (PPE) is imperative. However, the widespread shortage of PPE is well known, which requires a degree of ingenuity to tap into other meaningful resources [2] and to close the gap between the need for and the availability of PPE. The U.S. Centers for Disease Control and Prevention has itself considered the use of nonmedical devices in crisis situations [3].

The severe acute respiratory syndrome coronavirus 2 (SARS-CoV-2) has been extensively analyzed, showing that it is sustained in aerosols for up to 3 hours and for up to 72 h on plastic and stainless steel surfaces after contamination. Even though research shows an exponential decay in the virus titer in all experimental settings [4], it also reveals that high viral loads in the upper respiratory tract might be a factor in its epidemiologic characteristics. Furthermore, the potential for people infected with SARS-CoV-2 to transmit the virus while asymptomatic [5,6], the mode of transmission, and the aerosol and surface stability of SARS-CoV-2 require a specific focus on preventive measures [7], which may vary depending on the activities that HCWs perform [8].

There is currently no universal standard for face/eye protection from biological hazards, which is reflected in the diversity of public health care guidelines [8,9,10]. Face shields in combination with additional PPE (goggles and mouth/nose mask) have been shown to reduce the risk of inhalational exposure, especially when performing activities with aerosol formation [9,11]. HCWs, particularly those prone to contact with potentially infected aerosols, are at high risk of exposure to an increased viral load and should be provided with the appropriate PPE to prevent infection and avoid possible nosocomial spreading routes [12]. Face shields are PPE devices used by many workers (e.g., medical, dental, and veterinary personnel) for protection of the facial area from splashes, sprays, or the spatter of body fluids. However, face shields should be used with other protective equipment and, as such, are classified as adjunctive PPEs [9]. Recommendations on face shield use differ depending on the setting but the essential use of PPEs in the population during a pandemic has led the WHO to appeal for an increase in their production [13]. Supplementary PPEs are recommended for high-risk HCWs in particular, including goggles for eye protection and face shields to avoid contamination of the facial area. Due to their proximity to patients and subsequent potential exposure to infected airborne particles or aerosols [14,15], the presence of oral fluids, and the use of sharp instruments, HCWs are at increased risk of SARS-CoV-2 infection [16].

The quest to find alternative resources and solutions to overcome the shortage of PPE also suggests a relaxation of the less essential regulatory requirements. Face shields are considered Class I medical devices, exempt from Food and Drug Administration (FDA) Pre-Market Notification (Form 510 [K]) [9], which can be manufactured using materials that have been classified harmless when in contact with skin or food. Therefore, to provide HCWs with sufficient PPEs to increase facial protection, face shields could be 3-D printed. 3-D printers are in widespread use for professional and private purposes. Despite not being as fast as injection molding processes, this method of production allows the at-home, on-demand manufacture of face shields by a broad spectrum of users. This study evaluates the utilization of 3-D printers, which are otherwise used for dental purposes, to produce face shields using open-source design data and investigates their clinical suitability.

## 2. Materials and Methods

### 2.1. Face Shield Characteristics

**Figure 1:**
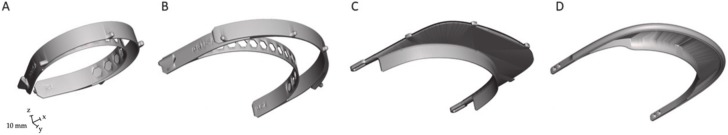
Open-source standard tessellation language (STL) datasets of (**A**) RC1, (**B**) RC2, (**C**) Budmen V3, and (**D**) Easy 3D Face Shields.

Four popular open-source standard tessellation language (STL) datasets for the frameworks of four face shields were selected (Figure 1): (A) RC1 (Prusa Research, Holešovice, Czech Republic, https://www.prusa3d.com/covid19) consists of a partially circumferential headframe with a clamp fit that is connected by a frame holding four integrated pins for the attachment of the visor. The area between the headframe and frame for the visor is open, with the connective area being positioned in the distal part of the headframe. (B) Compared to RC1, the material thickness of the RC2 (Prusa Research, Holešovice, Czech Republic) framework is greater. The basic design is similar to that of RC1 except the headframe does not have a clamp fit. (C) Budmen V3 (3D Face Shield V3, Budmen Industries, Philadelphia, PA, USA, https://budmen.com/) consists of one closed and stiffened frame (no clamp fit) extending outward with four pins to attach the visor. (D) Easy 3D (Easy 3D printed Face Shield, Hanoch Hemmerich, La Laguna, Tenerife, Spain, https://www.thingiverse.com/thing:4233193) consists of one frame (no clamp fit) with an extension for the visor that is fixed by clamping it into a slot.

### 2.2. Manufacturing

The data sets for the face shields were downloaded as standard tessellation language (STL) files and imported into a slicing software application (Prusa Slicer, Prusa Research; Holešovice, Czech Republic). A biodegradable material (DIN EN ISO 14855) with a melting temperature of >190 °C and a Vicat softening temperature (VST) of 160 °C was used as the filament for printing (GreenTEC PRO, 1.75 mm, Extrudr, Lauterbach, Austria) with a fused deposition modeling printer (Prusa i3 MK3S, Prusa Research, Holešovice, Czech Republic). Printing was performed on a powder-coated print bed at a printing temperature of 220 °C, a printing bed temperature of 60 °C, a printing speed of 40 mm/s, a nozzle size of 0.4 mm, 20% infill, and a layer height of 0.2 mm. Each face shield was printed individually.

For completion, a 0.1-mm thick polyethylene terephthalate (PET) DIN A4 (210 × 297 mm) transparent foil (Laser foils, Dots Office Products, Kansas City, MO, USA) was prepared and mounted to the frame as described: for the RC1, RC2, and V3 face shields, four holes were made in the visor at the intended locations using a DIN A4 perforator and then pressed onto the four pins for retention. For the Easy 3D Face Shield, the foil was clamped into a dedicated slot. At the posterior end of each frame, an elastic strap (0.6 mm, polyester, available by the meter) was attached.

### 2.3. Evaluation

After downloading and 3-D printing the STL datasets, the filament weight, total weight, printing time, and the necessary tools for assembling the framework were determined. For clinical assessment, 10 dental clinicians and/or intensive care nurses wore each face shield (n = 4) for one hour when performing dental treatment or intensive care. The clinical parameters examined were rated on a 10-cm visual analogue scale (VAS) ranging from 0 = “not acceptable” to 100 = “excellent” and measured with a ruler for subsequent evaluation. The parameters included the following: (1) the contact of the headframe with the forehead (fit), (2) comfort during usage, e.g., pressure on the head and slipping of the device (comfort), (3) sufficient space for additional protective devices, e.g., goggles or glasses and oronasal mask (space), (4) sealing of the forehead and lateral coverage of the cheeks and temporal/zygomatic area and the chin (protection), and (5) overall evaluation (overall).

### 2.4. Statistical Analysis

For analysis, the mean values and standard deviations were determined. To verify the normal distribution of the five parameters, the Shapiro-Wilk test was applied. Subsequently, a one-way analysis of variance (ANOVA) was applied, incorporating the five dependent variables (fit, comfort, space, protection, and overall), with the post-hoc Tukey test. The significance level was set at α = 0.05. The statistical analysis was performed using a statistical software (IBM SPSS Statistics, ver. 22.0, IBM Corp, Armonk, NY, US).

## 3. Results

### 3.1. Clinical Assessment

**Figure 2:**
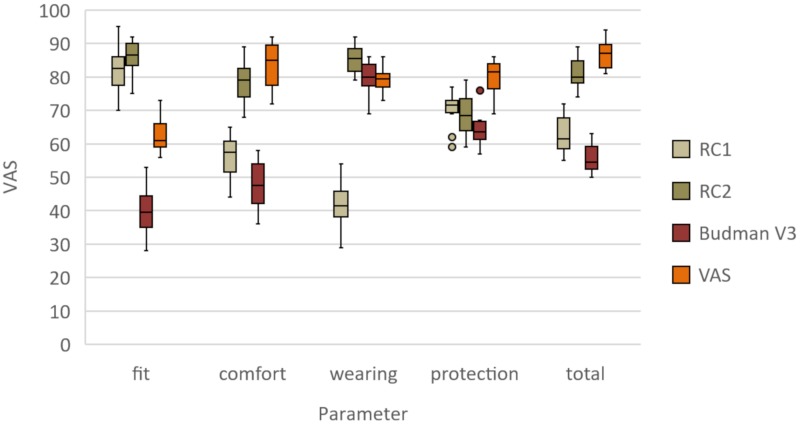
Boxplots of minimum, maximum, interquartile range, and median values and outliers of the evaluated parameters for the four open-source face shields.

The Shapiro-Wilk test revealed no significant differences (*p* ≥ 0.168). Therefore, normal distribution of the data was assumed. By contrast, one-way ANOVA showed significant differences for all five parameters (*p* = 0.001) (Figure 2). The fit was rated significantly better for RC1 (82 ± 8) and RC2 (86 ± 5) compared to that for Easy 3D (63 ± 6) (*p* = 0.001) and Budmen V3 (40 ± 8) (*p* = 0.001) (Table 1). Conversely, Easy 3D (83 ± 7) and RC2 (78 ± 7) displayed the highest wearing comfort compared to RC1 (56 ± 7) and Budmen V3 (48 ± 8) with significantly lower scores (*p* = 0.001). The space for additional protective devices was rated highest for RC2 (85 ± 5), showed no difference between Budmen V3 (80 ± 5) and Easy 3D (80 ± 4) (*p* = 0.997), and was rated significantly lower for RC1 (42 ± 8) (*p* = 0.001). In terms of protection, Easy 3D (84 ± 4) received the best rating, followed by RC1 (70 ± 6) and RC2 (69 ± 7), and, lastly, Budmen V3 (65 ± 6). Overall, Easy 3D (87 ± 4) and RC2 (81 ± 5) received the highest scores, which differed significantly from those for RC1 (63 ± 6) and Budmen V3 (56 ± 4) (*p* = 0.001).

### 3.2. RCI

The printing was possible without support structures, but the limited contact area with the print bed may cause detachment of the object during the printing process.

The size and the distance between the four integrated point attachments are designed to match the spacing of a perforator (DIN A4 standard), which makes assembly easy. An additional printed reinforcement with an incorporated slot helps keep the curvature of the foil on the lowermost side (shield curvature aid).

**Figure 3:**
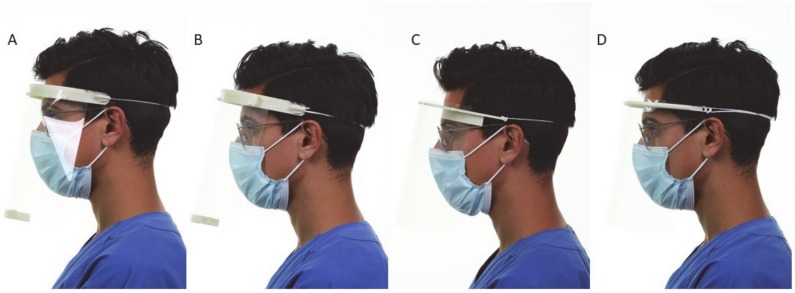
Profile view of the worn face shields showed limited space for additional PPE with (**A**) RC1, when compared with (**B**) RC2, (**C**) Budmen V3, and (**D**) Easy 3D.

The headframe has a clamp fit. However, this can become uncomfortable when worn for an extended period of time due to the pressure it exerts. The lateral expansion covers the cheeks and most of the zygomatic area. However, the space between the headband and the visor is limited. Consequently, eye and oronasal protective devices may interfere with the visor (Figure 3). Furthermore, the open space between the headframe and the visor frame does not provide protection against aerosols from above. Reinforcement of the lowermost side of the foil can be prone to error during printing (the narrow slot caused failures at high printing speeds or when using stereolithography-based printers) and may detach during treatments.

### 3.3. RC2

Similar to RC1, the printing of RC2 is possible without support structures, but the limited contact area with the print bed may cause detachment of the object during the printing process. RC2 is equipped with additional printed reinforcement at the lowermost side of the foil, as described for RC1.

The RC2 model does not have a clamp fit for the headframe, which increases the wearing comfort. However, the fit (the intimacy of the headframe contact with the forehead) remains similar to that with RC1. The increased space between the face and visor is sufficient for essential eye and mouth-nose protective equipment, but, at the same time, lateral protection against aerosols when using pre-fabricated DIN A4 foils is reduced. For some clinicians, the weight of the framework and the missing sealing between the headframe and visor (as with RC1) may be a disadvantage.

### 3.4. Budmen V3

The Budmen V3 face shield offers a simplified geometry with a higher contact area with the print bed, which minimizes printing failures and shortens the printing time.

The assembly is comparable to that of RC1 and RC2 (designed to match the spacing of a perforator of DIN A4 standard). The space available for additional PPE is comparable to that with RC2. The lateral expansion and complete sealing of the forehead provide more protection from above and laterally. The headband is rigid due to its one-piece construction impairing its completely intimate adaption to the head and minimizing its fit. The rigidity of the face shield reduces wearing comfort and fit compared to RC2.

### 3.5. Easy 3D

The contact area with the print bed is large, which makes detachment during printing unlikely. This process shortens the printing time. Conversely, the printing of the narrow visor slot might pose problems at high printing speeds, and the delicate design might be more prone to breakage.

After printing, the visor is pushed into a small continuous slot with clamping retention. This eliminates the need for additional steps, such as perforations. Furthermore, the visor remains stable and can be changed easily. The lower weight of the Easy 3D makes it comfortable to wear. The space beneath the visor is less than that with RC2 but more than that with RC1. The headframe fits snuggly to the forehead and offers good protection from aerosols since the forehead is sealed, and the lateral extension of the visor covers the cheek and the zygomatic areas.

## 4. Discussion

### 4.1. Rationale

The present article is based on the belief that documentation and transparency [17] using open-source data will help identify ways to combat the spread of SARS-CoV-2 infection. The rationale of the present investigation was to remove the necessity of time-consuming testing procedures with respect to printing time, ease of assembly, printability, and the comfort of using open-access data to print face shields in situations of scarcity. Similar in design to commercially available face shields, the samples presented in this study represent a simplified design that allows for on-demand manufacturing of face shields and assembly using common house-hold items.

### 4.2. Printing Technique

For effective and efficient manufacturing of the face shields presented in this study, fused deposition modeling (FDM) was preferred due to its increased printing capability and minimal post-processing requirements. By contrast, stereolithography (SLA) printers require uncured resin to be removed from the finished print using cleaning or washing procedures prior to additional light-curing [18,19]. While both printing techniques require support structures in non-supported areas, FDM-based techniques will generally require fewer support structures. Thus, this process further decreases the time needed for post-processing. Moreover, some design features such as the clamping slot of the Easy 3D Face Shield or the shield curvature aid of the RC2 can result in failures when using SLA-techniques due to the non-removable support structures in non-accessible gaps or fissures.

### 4.3. Printer Requirements

Besides the previously mentioned considerations regarding printing technology, printer selection for present purposes depends on the print volume requirement needed to meet the size of the printed dataset (Table 1). In the present case, the Prusa RC2 design was selected as a reference, requiring a width of 144 mm, a length of 191 mm, and a height of 20 mm. The Prusa i3 MKS3 sample used in the present study features a print bed with a width of 210 mm, a length of 210 mm, and a maximum printing height of 250 mm. The sample is, therefore, suitable for printing all of the face shields evaluated. Other printers, e.g., Form 3 (Formlabs, Somerville, Massachusetts, USA) do not offer the printing volume needed. In order to print the RC2 design, the dataset would have to be scaled to 95% and/or the printing direction changed to fit onto the printing platform. Changing the size may result in an improved or diminished fit of the face shield. Moreover, the benefit of a support-structure free design is no longer present when longer printing times are needed due to having to change the printing direction.

### 4.4. Material Selection

Acrylonitrile butadiene styrene (ABS) features good strength but not biodegradability. In addition, ABS has to be printed at higher temperatures (240–250 °C) compared to polylactide (PLA, 180–220 °C) and is, therefore, more susceptible to shrinkage during cooling. As a consequence, ABS is considered to be more prone to failures during the printing process. Polyethylene terephthalate (PETG) combines both the high fracture strength of ABS and the reduced susceptibility to failure of PLA. However, again, it lacks biodegradability. The material used in the present study is based on lignin (GreenTEC PRO) and combines the previously mentioned attributes [20]. Visors can be manufactured from several types of materials that include polycarbonate, propionate, acetate, poly(vinylchloride), and PETG [9]. Since PLA and PETG are known to show volume change during sterilization processes [21], one-time use of the visor is recommended, whereas a study showed that sterilized 3-D printed surgical splints using filaments based on lignin (Green-TEC Pro) remained dimensionally stable [22]. The sterilization of the frame might be possible, but was not tested in this study. Therefore, a recommendation regarding sterializability cannot be made. The biodegradation of the material, documented according to the EN 13432 standard, will minimize the impact of plastic on the environment.

### 4.5. Assembly and Protection

The lack of requirement for a DIN A4 perforator can be considered to be a major advantage of the Easy 3D face shield design, allowing assembly without the need for further region-specific (DIN A4) tools. The small radius of curvature of the Easy 3D visor compared to that of the RC1/RC2/V3 models enables both the space for expansive PPE, such as goggles, as well as extended lateral protection against aerosols. However, even though the lateral extension of the visor was rated best compared to that of the other frames, the recommendation of extension to the ear was still not met [9]. This can be overcome by customizing the size of the PET foil.

### 4.6. Scalability

Mass production using non-industrial in-office 3-D printers might be limited due to their printing times, which were greater than 1.40 h. In a more recent version of the RC2 design, print files with four frames stacked on top of one another were provided by the developers (RC3). Stacking frames may be an easy method for overnight printing in a private, non-industrial setting. Other frames evaluated in the current study, such as the Easy 3D face shield, cannot be stacked in the same way without additional support structures due to the closed one-piece design with a protruding frontlet. Thus, its cost-effectiveness remains debatable. From a scalability point of view, the RC3 is recommended for mass production in non-industrial environments. However, the Easy 3D frame is a viable option for small scale in-house production.

In general, open-source data with a limited need for support structures are preferred due to their printing speed and economic efficiency (waste of material) [23]. Most of the available online datasets, such as that for the production of PPE including respirators, were not specifically designed for additive manufacturing. Such datasets might be used for the private production of prototypes but cannot be recommended for large-scale production during times of material shortages.

### 4.7. Future Developments

Easy to use and self-sustaining face shield production using one’s own printing capabilities in combination with sufficiently available, commercial goods, such as the laser copy foils in the current study, can be considered to help protect HCWs from aerosol or accidental hand-to-face contamination during their daily clinical routines. PET foils for visors should be individually sized to full facepiece length that extends below the chin and offers sufficient width to reach at least the point of the ear for more peripheral protection. Customization of the available datasets for face shield production on the basis of factors such as 3-D face scans might improve wearing comfort, fit, and sealing capacity and increase protection. However, this may require time-consuming individual adaptation of the datasets.

In general, the ongoing pandemic might result in the rethinking of material supply for healthcare institutions. In particular, increased efforts in realizing real-time, on-demand in-campus production allows for independence from external suppliers and a decreased need for stock-piling [24]. This will increase flexibility in daily clinical routines and autonomy in emergency situations but will introduce new regulatory challenges [25]. Printing ready-to-use sterile parts using specific cleanroom printing devices will enable further optimization of just-in-time production in the near future and further broaden the application range of additively manufactured goods in hospitals [26].

Though the face shields produced for the current study can reduce the viral load on the facial area from aerosol penetration, they should not be used as solitary face/eye protection, but rather as an adjunct to other PPE such as protective eye/nose masks and goggles.

## 5. Conclusions

3-D printing of the frame of a face shield was possible using open-source data and biodegradable material. When printing face shields, the printing ability and printing time need to be considered and may vary depending on the design. For clinical suitability, a design should be chosen to allow sufficient space for goggles and N95 respirators as well as maximum coverage of the facial area. Due to its printability, ease of assembly, space for additional PPE, and protection, the Easy 3D face shield was shown to be the most effective. However, it lacks scalability potential (stacking) in non-industrial environments. If large-scale production is a priority, the RC2 should be the preferred option due to the availability of a stacked dataset.

## Figures and Tables

**Table 1:** Evaluated parameters of 3-D printed open-source face shields.

Name	RC1 (Prusa)	RC2 (Prusa)	3D Face Shield V3 (Budmen Industries)	Easy 3D Face Shield (Hanoch Hermann)
**Print volume requirement**	135 × 120 × 20 mm	144 × 191 × 20 mm	141 × 187 × 20 mm	146 × 165 × 15 mm
**Filament weight**	30 g	42 g	33 g	21 g
**Total weight**	39 g	51 g	42 g	30 g
**Printing time**	2 h 30 min	3 h 17 min	2 h 06 min	1 h 40 min
**Tools for** **assembling**	DIN A4 perforator	DIN A4 perforator	DIN A4 perforator	No
**Fit**	82 ± 8	86 ± 5	40 ± 8	63 ± 6
**Comfort**	56 ± 7	78 ± 7	48 ± 8	83 ± 7
**Space for additional PPE**	42 ± 8	85 ± 5	80 ± 5	80 ± 4
**Protection**	70 ± 6	69 ± 7	65 ± 6	84 ± 4
**Overall**	63 ± 6	81 ± 5	56 ± 4	87 ± 4
